# Screening for diabetes and hypertension in adult dental patients: the experience in a Nigerian dental center

**DOI:** 10.3389/fdmed.2024.1468375

**Published:** 2024-12-18

**Authors:** Ukachi Chiwendu Nnawuihe, Emmauel Adetolu Adelusi, Afolake Shakirat Salami, Ezekiel Taiwo Adebayo, Lilian Ejije Ahaji

**Affiliations:** ^1^ Department of Public Health, Intercountry Centre for Oral Health for Africa, Jos, Nigeria; ^2^ Department of Oral and Maxillofacial Surgery, University of Medical Sciences, Ondo, Nigeria

**Keywords:** hypertension, diabetes mellitus, screening, adult dental patients, Nigeria

## Abstract

**Objectives:**

The aim of the present study was to examine the presentation of hypertension and diabetes mellitus in dental patients.

**Methods:**

Dental patients were screened for hypertension and elevated blood sugar using a sphygmomanometer and a glucometer, respectively.

**Results:**

A total of 102 men and 129 women participated in the study. In total, 69 (29.9%) and 20 (8.7%) participants reported a history of hypertension and diabetes mellitus, respectively. Adherence to taking hypertensive and diabetic medications as advised by a clinician was reported by 68.0% and 85.0% of patients with known hypertension and diabetes, respectively, of whom 29.8% had uncontrolled hypertension and 29.4% had high blood sugar in the diabetic range (>200 mg/dl). In patients with no history of these diseases, 42 (25.9%) had elevated blood pressure, while 5 (2.4%) had high blood sugar in the diabetic range. The prevalence of hypertension was 37.3% and high blood sugar in the diabetic range was 5.2% in the sample. Individuals with diabetes were 31 times more likely to have hypertension than those without diabetes (odds ratio = 31.06, 95% confidence interval 5.68–169.98, *p* ≤ 0.001).

**Conclusion:**

Screening was helpful in the detection of undiagnosed cases and suboptimal control of both diseases in patients. Recommendations for dental practice guidelines include routine screening with mandatory screening for hypertension in patients with diabetes.

## Introduction

Diabetes mellitus is a chronic metabolic disease characterized by elevated levels of blood glucose (or blood sugar), which, over time, leads to serious damage to the heart, blood vessels, eyes, kidneys, and nerves (1) while hypertension is a sustained systemic blood pressure >140/90 mmHg (2). Both conditions can occur in patients as isolated medical conditions and can coexist with or without other systemic diseases (1, 2).

Hypertension and diabetes combined are responsible for nearly 13 million preventable deaths globally (3, 4). The majority of these deaths and approximately 75% of the burden occur in low- and middle-income countries, a group that encompasses the majority of African countries, including Nigeria (3–5). In addition to the reported preventable mortality and morbidity that make both diseases a public health concern, there is documented evidence of increasing prevalence attributed to sedentary lifestyles, tobacco and alcohol consumption, and diets rich in refined sugars and salt (1–5).

The association of both conditions with increasing age and female predilection has also been observed (6, 7). The association between increasing age, female gender, hypertension, and diabetes mellitus has been attributed to age-related physiological decline and hormonal changes in post-menopausal women (1, 2, 8).

Hypertension and diabetes mellitus are reported to be common in the Nigerian adult population (1, 3, 9–14). A significant number of affected adults are unaware of these diseases, as both hypertension and diabetes mellitus tend to manifest in an insidious manner (1, 3, 15). Early diagnosis and intervention for both diseases are highly desirable, as lifestyle modifications may be the only prescribed treatment for disease control (3–5, 15). Hypertension and diabetes mellitus have been reported among adult dental patients (3, 10), with a documented prevalence of hypertension and diabetes mellitus ranging from 20% to 45% (6–12) and 10%–25%, respectively (6, 13, 14).

Hypertension and diabetes mellitus have been linked to compromised oral health (13, 14). Uncontrolled hypertension and diabetes mellitus, along with associated risk factors, can initiate or worsen oral health issues. Both conditions pose significant risks not only to the oral health of patients but also to the management of oral diseases/conditions, due to their adverse effects on multiple body systems (7, 10, 13, 15). Opeodu and Adeyemi (6) reported that patients with hypertension and diabetes mellitus are more susceptible to dental treatment complications such as syncope, infection, and delayed or poor wound healing. Despite the high prevalence in the adult population and the benefits of early detection, intervention, and awareness of risks to oral health and dental management, many dental clinics in Nigeria lack established protocols or guidelines for early detection of elevated blood pressure and blood sugar, both of which are major indicators of hypertension and diabetes mellitus (1, 9, 10, 15). This highlights the need to assess the likelihood of adult patients with these conditions presenting to dental clinics. These data can support the development of the aforementioned protocols and practice guidelines to improve intra- and postoperative patient outcomes in addition to facilitating early detection and intervention of hypertension and diabetes mellitus. This rationale informs the reason for this study, which aimed to examine the incidence of these conditions among adult patients attending a tertiary oral care center in Nigeria.

## Materials and methods

### Study setting and design

This cross-sectional study was conducted at the dental center of the University of Medical Sciences Teaching Hospitals, Ondo City, Ondo State, Southern Nigeria, among adult patients attending the center between May 2023 and April 2024.

The minimum sample size for this study was determined using the Araoye formula (16)

$$n=\frac{t^{2}\times p(1-p)}{m^{2}}$$

where *n* = minimum sample size, *t* = standard normal deviation at 95% confidence level (standard value of 1.96), *p* = estimated prevalence of hypertension in the Ondo State, Nigeria (18.2%) (13), and *m* =  5% margin of error (standard value of 0.05). The minimum sample size required for the study was 229. Ethical approval for the study was obtained from the University of Medical Sciences Teaching Hospitals’ Ethics and Research Committee (Ethical approval reference number: UNIMEDTHC/024/ERC/122).

### Methods

#### Procedure

Information on age, gender, hospital number, date of birth, occupation, and level of education attained was collected from adults aged ≥18 years who provided informed consent through interviewer-administered questionnaires. A medical history of hypertension, diabetes mellitus, and medication adherence (if positive for either condition) was also recorded. Then, each participant’s blood pressure and random blood sugar readings were taken and documented.

An aseptic technique was maintained while measuring blood pressure and blood sugar levels. Blood pressure readings were taken after a 10-min rest period, with the participants seated. Measurements were taken from the upper part of the non-dominant arm using a sphygmomanometer and stethoscope. Blood pressure was measured a second time, 5 min later. The average of the first and second readings was recorded as the participant's blood pressure. If the difference between the two readings was >10 mmHg, a third measurement was taken 5 min later. The average of the three measurements was recorded as the participant's blood pressure.

For the random blood sugar test, participants were asked to wash their hands with soap and running water, and then dry them with a clean napkin. A 70% alcohol swab was used to clean the thumb, which was then pricked with a lancet to collect a drop of blood. The blood was placed on a test strip and inserted into the glucometer. Random blood glucose values were recorded from the capillary blood using a FineTest® glucometer (Auto-coding™ Premium, Infopia Co. Ltd., Republic of Korea). If the value was above 110 mg/dl, the patient was recalled the next day after at least 8 h of fasting for a repeat test.

The primary outcome variables were diabetes mellitus and hypertension. Hypertension is categorized as <120/80 mmHg (normal), 120–139/80–89 mmHg (pre-hypertension), 140–159/90–99 mmHg (stage I hypertension), and >160/100 mmHg (stage II hypertension) or previously diagnosed hypertension on medication (13). In this study, diabetes mellitus is defined as random blood sugar >200 mg/dl, fasting blood sugar >126 mg/dl, or previously diagnosed diabetes on medication (13). Patients with uncontrolled or newly detected hypertension, and those with elevated blood sugar above 110 mg/dl, were referred to the physician for a full clinical evaluation and treatment.

#### Data collection and analysis

The anonymity of the participants and protection of the data was guaranteed by storing research data on a personal computer with a password known only to one researcher. Data were extracted into Microsoft Excel and checked for completeness by double entry, including random checks for errors and outliers. These data were then transferred to SPSS version 25.0 (IBM Corp., Armonk, NY, USA) for statistical analysis (17). Categorical variables were expressed as frequencies and percentages, while numerical variables were expressed as means. The chi-square and Fisher’s exact tests were used to test for relationships between categorical variables. The correlation between blood pressure, blood sugar, and demographic data was assessed using Pearson's correlation coefficient. Logistic regression was used to determine the risk of identifying diabetic and hypertensive patients in the dental clinic. A 95% confidence interval was used to measure the precision of the estimates, with statistical significance set at *p* < 0.05.

## Results

A total of 231 patients [102 men (44.2%), 129 women (55.8%); mean age 48.3 ± 17.5 years; age range 18–96 years] participated in the study. Among the participants, 154 (66.7%) worked in the informal sector, while 77 (33.3%) worked in the formal sector.

In the present study, 69 (29.9%) participants reported a medical history of hypertension, and 47 of them(68.1%) reported that they were compliant with their blood pressure regulation medication. In addition, 20 (8.7%) participants reported a medical history of diabetes mellitus and 17 of them (85.0%) were compliant with their blood sugar regulation medication. Among the participants, 16 out of 20 (80.0%) individuals who reported a medical history of diabetes mellitus also reported a medical history of hypertension. The association between medical history of hypertension and diabetes mellitus was significant (*p* ≤ 0.001) (Table 1).

**Table 1:** The association between history of hypertension and diabetes mellitus.

	History of DM		Total *n* (%)	χ^2^	*p*
	Yes *n* (%)	No *n* (%)			
History of HBP					
Yes	16 (6.9)	53 (22.9)	69 (29.9)	26.267	<0.001^a^
No	4 (1.7)	158 (68.4)	162 (70.1)		
Total	20 (8.7)	211 (91.3)	231 (100.0)		

HBP, high blood pressure; DM, diabetes mellitus.

^a^ Statistically significant.

Among the 47 individuals who reported being compliant with their blood pressure regulation medication, 14 (29.8%) had elevated blood pressure. Among the 17 individuals who reported being compliant with their blood sugar regulation medication, 5 (29.4%) had elevated blood sugar above the reference value for diabetes mellitus.

In this study, of the 162 patients without a history of hypertension, 42 (25.9%) had elevated blood pressure [pre-hypertension = 8 (19.0%), stage I hypertension = 28 (66.7%), and stage II hypertension = 6 (14.3%)]. Of the 211 individuals without a history of diabetes mellitus, 5 (2.4%) had diabetes mellitus.

Of the participants, 135 (58.4%) had normal blood pressure. The prevalence of hypertension was 37.3% [stage I and II hypertension were found in 63 (27.3%) and 23 (10.0%) patients, respectively]. The prevalence of diabetes mellitus was 5.2%. The association between blood pressure and diabetes mellitus is significant (*p* ≤ 0.001) (Table 2).

**Table 2:** The association between blood pressure and diabetes mellitus.

	Diabetes mellitus		Total *n* (%)	χ^2^	*p*
	No *n* (%)	Yes *n* (%)			
Blood pressure					
Normal	130 (56.3)	5 (2.2)	135 (58.4)	4.536	<0.001^a^
Pre-stage hypertension	9 (3.9)	1 (0.4)	10 (4.3)		
Stage I hypertension	57 (24.7)	6 (2.6)	63 (27.3)		
Stage II hypertension	23 (10.0)	0 (0.0)	23 (10.0)		
Total	219 (94.8)	12 (5.2)	231 (100.0)		

^a^ Statistically significant.

Stage II hypertension and diabetes mellitus were more common in men and the two upper age groups. Although there is no significant association between gender and either condition, there was a significant association between increasing age and hypertension observed in this study (Tables 3, 4).

**Table 3:** The association between blood pressure with age group and gender.

	Blood pressure				Total *n* (%)	χ^2^	*p*
	Normal *n* (%)	PSH *n* (%)	S-I H *n* (%)	S-2 H *n* (%)			
Age group (years)							
18–39	74 (32.0)	2 (0.9)	8 (3.5)	0 (0.0)	84 (36.4)	55.871^a^	<0.001^b^
40–64	43 (18.6)	5 (2.2)	37 (16.0)	17 (7.4)	102 (44.2)		
≥65	18 (7.8)	3 (1.3)	18 (7.8)	6 (2.6)	45 (19.4)		
Gender							
Male subjects	56 (24.2)	3 (1.3)	30 (13.0)	13 (5.6)	102 (44.2)	2.882	0.424
Female subjects	79 (34.2)	7 (3.0)	33 (27.3)	10 (10.0)	129 (55.8)		
Total	135 (58.4)	10 (4.3)	63 (27.3)	23 (10,0)	231 (100.00		

PSH, pre-stage hypertension; S-I H, stage I hypertension; S-2 H, stage II hypertension.

^a^ Fisher’s exact test.

^b^ Statistically significant.

**Table 4:** The association between diabetes mellitus with age group and gender.

	Diabetes mellitus		Total *n* (%)	χ^2^	*p*
	No *n* (%)	Yes *n* (%)			
Age group (years)					
18–39	82 (35.5)	2 (0.9)	84 (36.4)	2.695	0.317
40–64	96 (41.6)	6 (2.6)	102 (44.2)		
≥65	41 (17.7)	4 (1.7)	45 (19.4)		
Gender					
Male subjects	96 (41.6)	6 (2.6)	102 (44.2)	0.175	0.448
Female subjects	123 (51.3)	6 (2.6)	129 (55.8)		
Total	219 (94.8)	12 (5.2)	231 (100.00		

Logistic regression was performed to determine the effect of diabetes mellitus, age, and gender on the presence of hypertension. Table 5 shows these results. The model was statistically significant (χ^2^_(5)_ = 45.486, *p* ≤ 0.001). The model correctly classified 83.9% of the cases, and 30.0% of the variance in hypertension was explained, as indicated by the Nagelkerke *R*^2^. Participants who were diabetic were 31 times more likely to be hypertensive than non-diabetic participants. Patients who were aged 65 years and older were at least 12.5 times more likely to have hypertension than the youngest age group (18–39 years). Patients who fell into the middle age group (40–64 years) were twice as likely to have hypertension as the youngest age group. However, this association is not significant (*p* = 0.078) (Table 5).

**Table 5:** Logistic regression analysis for the relationship between hypertension with age and diabetes mellitus.

Patients’ characteristics	*P*	OR	95% CI for OR
Age group (years)			
≥65	<0.001	12.53	3.462–45.380
40–64	0.078	2.13	0.918–4.957
18–39		1.00	
Diabetic			
Yes	<0.001	31.06	5.675–169.982
No		1.00	

CI, confidence interval; OR, odds ratio.

## Discussion

This study screened for hypertension and diabetes mellitus among adults attending the dental center of a Nigerian tertiary hospital. The majority of participants worked in the informal sector, which is not surprising as the informal sector is the largest employer of labor in Nigeria, engaging approximately 60% of the working population (18). A higher proportion of women attended the hospital in this study, which is consistent with similar findings in the literature (6, 13, 14). One reason for this finding is that women have better health-seeking behavior.

The prevalence of hypertension (37.3%) and diabetes mellitus (5.2%) observed in this study is within the range of prevalence of hypertension (18.2%–39.9%) and diabetes mellitus (4.4%–6.3%) reported in previous Nigerian studies (6, 9, 10, 12, 19). Although a greater proportion of participants with a history of diabetes mellitus (85.0%) reported greater medication adherence than participants with hypertension (68.1%), nearly one-third of the participants who reported medication adherence for both systemic conditions had elevated blood pressure and blood sugar above the reference values for hypertension and diabetes mellitus. Greater medication adherence among individuals with diabetes in this study may stem from a greater appreciation of their health, as many of them also have hypertension. Hence, they are better motivated to safeguard their health. Other contributing reasons for this observation include poor adherence to certain antihypertensive medications due to their side effects (20). However, the reasons for non-adherence were not explored in this study. A key finding was that nearly one-third of patients who reported medication adherence still had uncontrolled blood pressure and blood sugar. This underscores the importance of not taking patients’ reports at face value when taking their medical history. Some patients may not be adhering to their prescribed disease management regimen. This finding calls for dentists to be diligent when reviewing patients with a history of hypertension or diabetes mellitus to verify that patients’ reported health behaviors align with their clinical status.

This study highlights the usefulness of routine screening for blood pressure and blood sugar in adult dental patients, as 25.9% and 2.5% of patients were found to have hypertension and high blood sugar, suggestive of diabetes mellitus, respectively. Oral diseases are linked to these conditions (1, 6), and dietary and lifestyle behaviors that increase the risk of both diseases have also been linked to dental caries, periodontal disease, and oral cancer (6). Identification and control of hypertension and diabetes mellitus may facilitate early resolution of these oral conditions. Early diagnosis is an additional benefit of screening. The importance of early diagnosis and prompt intervention for these conditions cannot be overemphasized due to their insidious nature in causing damage to multiple systems in the body (3, 4, 14, 15). In addition, early diagnosis may provide a route to cost-effective management of the conditions, focusing only on lifestyle modifications instead of medications (3, 4, 14).

According to Pinto (8), physiological age-related changes in the human circulatory system, such as the thickening of the connective tissue and progressive impairment of blood pressure-regulating neurosensory receptors of the vascular smooth muscles, increase peripheral arterial resistance. In addition, body fluid volume increases due to decreased intercapillary interchange of fluids (11, 20). This may explain why hypertension is associated with increasing age in this study. This study finding aligns with reports by Bello-Ovosi et al. (15) and Uloko et al. (1), in their studies of hypertension and diabetes mellitus in the Nigerian population. Stress reduction techniques for elderly dental patients should be a routine practice, and the use of adrenaline-containing local anesthesia should be applied with caution due to the increased risk of elevated blood pressure.

Among study participants, the risk of hypertension doubles in middle age and is significantly higher in the elderly. In addition, participants with diabetes are at a relatively higher risk of developing hypertension compared with non-diabetic patients. Diabetes mellitus adversely affects cellular communication, immune response, neurosensory mechanisms, collagen formation, and the remodeling of the peripheral blood system (1, 13). These factors may contribute to the increased risk of hypertension in diabetic patients. This finding is significant when designing a screening protocol for this population. Therefore, routine screening for hypertension should be encouraged in middle-aged adults in the study population. This will contribute to the reduction of the unawareness associated with hypertension in the Nigerian population, with early detection and intervention providing benefits in the form of the adoption of healthy lifestyle changes (11). Furthermore, elderly individuals and diabetic patients should be screened for hypertension during dental clinic visits.

No association was found between gender and either condition in this study. Although a gender predilection for hypertension and diabetes mellitus has been reported in the literature (7), factors such as the influence of female hormones on fat deposition have been suggested to contribute to the increased risk of diabetes mellitus in women by Peters and Woodward (7). In addition, unhealthy lifestyles and poor health behaviors have been blamed for the higher prevalence of hypertension in men in previous studies (3, 4, 10). The reason for the parity of the association between gender and both conditions in this study is unknown. However, it is possible that the dominance of socioenvironmental factors over genetic factors may account for this outcome (21).

The findings from this study acknowledge the vital role that oral health teams play in making the dental clinic setting a vital place in the course of detection and management of both hypertension and diabetes mellitus. However, the Nigerian oral health system is characterized by grossly inadequate funding, access to oral healthcare, and uptake (22). Taking the aforementioned issues into account against the background of the high burden of preventable deaths and increasing prevalence associated with hypertension and diabetes mellitus (3–5), this calls for the urgent inclusion of oral health promotion efforts in a multidisciplinary integrated strategy for early detection, control of risk factors, and better management outcomes for both conditions. This integrated multidisciplinary strategy will incorporate elements of the common risk factor approach to optimize the use of health resources efficiently in resource-constrained settings like Nigeria. By addressing hypertension, diabetes mellitus, and other diseases associated with identified risk factors, this strategy aims to drive efforts to improve oral health behaviors and increase access to oral healthcare services in a sustainable manner (22, 23).

A limitation of the present study is that he reasons for poor drug adherence among study participants were not explored. Furthermore, this is a single-center study, and there may be variations in multicenter studies involving larger sample sizes. Findings from such studies will certainly impact the precision of the findings, as the characteristics of the sample would more closely reflect the study population. However, the adequate sample size and meticulous data extraction process in this study will buffer potential deviations that may arise in multicenter studies with larger samples (24).

## Conclusions

The prevalence of hypertension and diabetes mellitus in adults attending dental clinics reflects the reported prevalence of both conditions in the Nigerian population. Routine screening of adult dental patients for hypertension and diabetes mellitus may be useful in the detection of undiagnosed cases and in identifying suboptimal drug adherence in those with a positive medical history. It is recommended that dental practice guidelines and clinical operational protocol designs include routine screening for hypertension and diabetes mellitus, with a special focus on middle-aged and older adult patients. Patients with diabetes must be routinely screened for hypertension. Large-scale studies are needed to refine these recommendations and provide baseline data to advocate for an integrated multidisciplinary approach aimed at better control of both diseases, while also enhancing the role of dental clinics in managing these conditions through improved access to and uptake of oral healthcare.

## Data Availability

The original contributions presented in the study are included in the article/Supplementary Material, further inquiries can be directed to the corresponding author.
